# Supplementary material for: Barriers in the access, diagnosis and treatment completion for tuberculosis patients in central and western Nepal: A qualitative study among patients, community members and health care workers
Source: PLoS One. 2020 Jan 15;15(1):e0227293. doi: 10.1371/journal.pone.0227293 (PMC6961875; doi:10.1371/journal.pone.0227293)
Supplement: Attachment — Submitted filename: RESPONSE TO REVIEWERS COMMENTS.docx [file pone.0227293.s001.docx]

5^th^ December 2019

Dear editor and reviewers,

We are very grateful for your evaluation of the manuscript. We noticed that you had a small note/recommendation for us.

Below, we have added our response with relevant corresponding changes in the manuscript.

We look forward to your kind consideration.

Sincerely yours,

On behalf of co-authors,

Bipin Adhikari

Comments to the Author

**Reviewer #4: Dear authors,

thank you for the opportunity to re-review the mansucript „Barriers in the access, diagnosis and treatment completion for Tuberculosis patients in central and western Nepal: a qualitative study among patients, community members and health care workers”.

I have only a small note:

Please delete the information on gender and age in the in-vivo codes.**

AUTHORS: Thank you very much for this suggestion. We have removed the information on age and gender below each quote within the revised manuscript.
